# Suppressive Effect of Huzhentongfeng on Experimental Gouty Arthritis: An *In Vivo* and *In Vitro* Study

**DOI:** 10.1155/2019/2969364

**Published:** 2019-12-04

**Authors:** Zi-cong Wu, Qiang Xue, Zhen-ling Zhao, Peng-jun Zhou, Qun Zhou, Zhen Zhang, Jian-ping Deng, Ke Yang, Hua Fan, Yi-fei Wang, Zhi-ping Wang

**Affiliations:** ^1^Guangdong Provincial Engineering Center of Topical Precise Drug Delivery System, Department of Pharmaceutics, Guangdong Pharmaceutical University, Guangzhou 510006, China; ^2^Guangzhou (Jinan) Biomedical Research and Development Center, Guangzhou 510632, China; ^3^Chenguang Biotech Group Co., Ltd., Handan 056000, China; ^4^College of Life Science and Technology, Jinan University, Guangzhou 510632, China; ^5^Huazhong University of Science and Technology, Wuhan 430074, China

## Abstract

**Background:**

Huzhentongfeng (HZTF) is an extract from four Chinese medical herbs for treating gout. This study aims to evaluate its antigout activity and preliminary explore its mechanism *in vivo* and *in vitro*.

**Methods:**

The rats were intragastrically administered with HZTF for 5 days and then injected 0.1 ml (10 mg) of MSU crystals to their joints for generating a gout model to analyze the paw volume and histopathology of joint synovial tissues of rats with different doses. We also investigated the antioxidant capacity of HZTF *in vitro* using indication including lipid peroxidation, DPPH^·^, and ABTS^+^ radical-scavenging capacity; besides, we used qRT-PCR to measure the effect of HZTF on interleukin (IL)-1*β*, caspase-1, NLRP3, and NQO1 expression in hydrogen peroxide-stimulated RAW264.7 macrophages and IL-1*β*, IL-6, and tumor necrosis factor (TNF)-*α* in MSU crystal-induced THP-1 monocytes. Confocal microscopy analysis was used to observe the dimerization of ASC adapter proteins. In addition, we also established quality standard of HZTF by using the high-performance liquid chromatography (HPLC) method.

**Results:**

HZTF could significantly suppress the paw swelling and neutrophil infiltration induced by MSU intra-articular injection in rats compared with the control group. HZTF also showed inhibition effects of inflammatory cytokines (IL-1*β*, IL-6, and TNF-*α*) secretion at 25.00 and 50.00 *μ*g/ml in MSU-induced THP-1 cells but showed no effects of IL-1*β*, IL-6, and TNF-*α* mRNA expression in MSU-induced THP-1 cells. Furthermore, confocal microscopy analysis showed that HZTF could prevent the oligomerization of ASC. Moreover, HZTF also showed effects in cell-free and cell-base tests of antioxidant capacity.

**Conclusion:**

The results prove that HZTF possessed the potential preventive effect against gout arthritis, and the effect may be attributed to its preventing effect on neutrophil infiltration and proinflammatory cytokines secretion such as IL-1*β*, IL-6, and TNF-*α* which were caused by the activation of inflammasome.

## 1. Introduction

Gout is a common type of arthritis caused by hyperuricaemia and subsequent accumulation of monosodium urate crystal deposition in the joints, tendons, and surrounding tissues. The signs and symptoms of gout include sudden and intense pain, redness, swelling, and stiffness in a joint of the big toe or in another joint, such as the ankle, knee, elbow, wrist, and finger [1]. At present, there are many medications in clinic, such as nonsteroidal anti-inflammatory drugs (NSAIDs), colchicine, or corticosteroids, used to treat the symptoms of gout attacks, prevent future flares, and reduce the risk of gout complications. But they all have some limitations. Over 80% of patients treated with colchicine have experienced abdominal pain before full clinical improvement. In addition, the side effects of NSAIDs are more common in the elderly. Therefore, it is critical to develop more effective agents with less adverse reactions.

HZTF is extracted from four Chinese medical herbs which are widely used in China: *Polygonum cuspidatum* Sieb.et Zucc, *Plantago depressa* Willd, *Ligustrum lucidum* Ait, and Vespae nidus. These four medicinal materials have been used for treating gout for centuries [2–11].

The dried root of *Polygonum cuspidatum* Sieb.et Zucc was known as “Huzhang” in China, as a traditional Chinese medicine, which was officially listed in the Chinese Pharmacopoeia. The compounds from *Plantago depressa* Willd have been demonstrated to have tremendous amount of pharmacological activities, including antioxidant, antibacterial, and anti-inflammatory activities [12, 13]. *Plantago depressa* Willd contains large amounts of polyphenols such as resveratrol, and it is considered one of the best sources of resveratrol because it contains higher amounts of this compound than other plants or fruits [14]. *Plantago depressa* Willd or Cheqiancao in Chinese is widely distributed and utilised around world, which has no significant toxicities in a regular dosage and has been used as medicinal plants for centuries [15, 16]. Currently, researches have shown that extracts from Plantain leaves exhibited antioxidant and antimicrobial activities. The above-mentioned bioactivities of *Plantain* leaves extracts are thought to be related to phenolic compounds and flavonoids [17]. *Ligustrum lucidum* Ait is a kind of evergreen shrub plant which can be found in Hu'nan, Jiangsu, Sichuan, and Zhejiang provinces in China. The mature fruits of this plant have a cardiac and roborant effect and known as a traditional herb medicine in China, which is also prescribed in Chinese Pharmacopoeia. Previous phytochemical investigations on *Ligustrum lucidum* Ait reported the isolation of secoiridoid glycosides, phenolic glycosides, and lignans [18, 19]. Beehive is the hive of *Parapolybia varia* Fabricius, a widely used traditional Chinese medicine. It is widely distributed in China and be harvested in autumn and winter. After removing the dead wasps, the hive is manufactured into a kind of traditional Chinese medicine by open-air drying. Because of its multiple pharmacological activities, including antivirus and anti-inflammatory beehive has been used in traditional Chinese medicine over thousands of years for treating various diseases, including malignant tumors, rheumatoid arthritis, lung diseases, skin disease, digestive and urinary disorders, and dental diseases [11, 20].

Huzhentongfeng (HZTF) is an extract developed by GuangZhou (Jinan) Biomedical Research and Development Center for treating gout. In order to obtain a good antigout effect, we combined the traditional Chinese medicine theory system with modern medical theory to extract these four medicinal materials to produce HZTF. According to the theory of traditional Chinese Medicine, *Polygonum Cuspidatum* is a monarch drug, *Plantain* is a minister drug, *Ligustrum* is an assistant drug, and Nidus Vespae is the guide drug. And, its main function is to treat acute gouty arthritis.

Firstly, in order to ensure the stability of product quality of HZTF, we also established a standard for the content of medicinal materials by using the HPLC method.

Synovial cells, monocytes-macrophages, and neutrophils will produce multiple cytokines, such as IL-1*β*, IL-6, and TNF-*α*, when they were stimulated by MSU crystals in the joints. Our study demonstrated that HZTF showed prominent effect on neutrophil infiltration and paw swelling in rats induced by intra-articular MSU injection. However, the concrete mechanism of HZTF preventing gouty arthritis remains unclear. So, in this study, we investigated the inhibition effects on expression levels of IL-1*β*, caspase-1, NLRP3, and NQO1 in hydrogen peroxide (H_2_O_2_)-induced RAW264.7 macrophages and expression and secretion of IL-1*β*, IL-6, and TNF-*α* from MSU crystal-induced THP-1 cells to investigate the mechanism of HZTF in treating gouty arthritis.

Moreover, to investigate whether the antigout effect of HZTF was induced by its antioxidation activity, we tested the antioxidant effects of HZTF using lipid peroxidation, 1,1-diphenyl-2-picryl-hydrazyl free radical (DPPH)-scavenging activity, and 3-ethylbenzthiazoline-6-sulfonic acid (ABTS) radical-scavenging activity *in vitro*.

## 2. Materials and Methods

### 2.1. Equipment

Stainless steel tape gauge, produced by Southwest China Tool General Plant of Guizhou Aviation Industry Group, China. The model of UV-visible spectrophotometer used in this experiment is UV2600 (Shimadzu, Japan), the model of the centrifugal machine is HC-2518 (Anhui USTC Zonkia, China), the real-time thermal cycler is CFX96 Touch (BioRad, USA), and the microplate reader is ELx808 (BioTek, USA).

### 2.2. Reagents

HZTF was produced by Yili Pharmaceutical Co., Ltd., Guangdong, China. Tongfengshu was produced by Qinghai Green Pharmaceutical Co., Ltd, Qinghai, China. Dexamethasone (DEX) and MCC950 were produced by Selleck, Shanghai, China. Phorbol-12-myristate-13-acetate (PMA), ascorbic acid (VC), ABTS, DPPH, AAPH, tris, ferrozine, ammonium thiocyanate, methanol, ethyl alcohol, hydrochloric acid, potassium peroxydisulfate, ferric chloride, sodium dihydrogen phosphate, disodium hydrogen phosphate, sodium phosphate, hydrogen peroxide, uric acid, sodium hydroxide, MTT, and potassium persulfate were all in analytical grade and obtained from Aladdin Industrial Corporation, Shanghai, China. PCR Mastermix and cDNA reverse transcriptase kit were obtained from Takara, Beijing China. TRIzol reagent was from Tiangen, Beijing, China. Gene specific primers were from Sangon, Shanghai, China. Elisa kits were purchased from neobioscience, Guangdong, China. Anti-ASC antibody was from Proteintech, Illinois, USA.

### 2.3. Quality Standard of HZTF by HPLC

Quality standard of HZTF was performed as described in an earlier report with slight modifications [21]. Briefly, HZTF was dissolved in 70% ethanol, and HPLC analysis was conducted using Poroshell 120 SB-C18 (4.6 × 100 mm, 2.7 *μ*m, Agilent, US). The chromatogram was monitored at wavelength 280 nm. The mobile phase consisted of A (0.1% formic acid) and B (acetonitrile) with an isometric elution as follows: A 16% and B 84%. The flow rate of the mobile phase was 1.0 ml/min, and the injection volume was 10 *μ*l.

### 2.4. Synthesis of MSU Crystals

MSU crystals were synthesized as described [22]. Briefly, 1.68 g of uric acid in 0.01 M NaOH was in a water bath heated to 70°C. Then, 0.5 M NaOH was added to maintain pH between 7.1 and 7.2, and the solution was incubated at room temperature with slow stirring and continuously for 24 h. The crystals were washed with absolute ethyl alcohol, freeze dried, and autoclaved.

### 2.5. Animals

Male Sprague-Dawley rats (200 ± 20 g) were purchased from the Experimental Animal Center of Henan Province, China. The animals were allowed to adapt to their environment for at least one week before initiation of the experiments. The animals were housed five per cage under a normal 12 h/12 h light/dark schedule. The animals were housed at room temperature (20 ± 2°C) with relative humidity (60 ± 5%) and were free access to a standard diet in the duration of the study. All studies were operated in accordance with the Institutional Animal Care Committee at the Experimental Animal Center of Henan Province and the China Council on Animal Care at the Experimental Animal Center of Henan Province.

### 2.6. MSU Crystal-Induced Inflammation in Rats

#### 2.6.1. Animal Gout Model and Drug Administration

The rats were randomly divided into six groups. Each group contained ten rats. Group I served as controls and were intra-articular injected in the right posterior foot with 0.1 ml physiological saline. In group II, gouty inflammation was induced by intra-articular injection of 0.1 ml (10 mg) of MSU crystal suspension into the right posterior foot. Groups III, IV, and V comprised MSU crystal-induced rats treated with HZTF (0.25, 0.50, and 1.00 g/kg body weight, respectively). Group VI comprised MSU crystal-injected rats treated with positive control Tongfengshu (a commercially available Chinese patent medicine for gout, 1.50 g/kg body weight). HZTF and Tongfengshu were fully dispersed in distilled water. All doses of the respective drugs were expressed as grams per kilogram body weight. Drugs were intragastrically administered for 5 d once daily and also administration at the 5th day 1 h before the MSU crystal injection. Isoflurane was used to anesthetize the rats, and then the MSU suspension injection was given.

#### 2.6.2. Assessment of Paw Swelling

The pathological degree was quantified by the swelling value of paw measured at 1, 2, 3, 4, 5, 6, and 7 h after MSU crystal injections. The degree of edema was expressed as paw swelling which was calculated by the following equation:

(1) $$\begin{array}{c} \text{foot swelling value}=b-a. \end{array}$$

Here, *a* is the foot volume before MSU crystal injection and *b* is the foot volume after MSU crystal injection.

#### 2.6.3. Histopathological Analysis

Rats were killed by cervical dislocation (7 h), and then the ankle joints of the right posterior foot of the rats were dissected and fixed in 10% neutral formaldehyde fixative for synovial tissue collection. After decalcification, the tissues were paraffin-embedded, sectioned, and stained with haematoxylin and eosin (H&E).

### 2.7. Cell-Free *In Vitro* Studies

#### 2.7.1. Lipid Peroxidation

The thiobarbituric acid (TBA) method was carried out according to the method described by Costa et al. [23–25], and modified according to our situation. Briefly, 1 mL of the different concentrations of HZTF or VC was mixed with 1 mL of 10% yolk homogenate in tris buffer (pH 8.0). Then, 100 *μ*l of 70 mM AAPH, 2.9 ml 20% acetic acid, and 3.0 ml 0.8% TBA solution were added. And, the mixture was boiled 60 min for full reaction; after cooling, adding 4.0 ml butanol, and fully extracting, the mixture was centrifuged at 2000 g for 10 min, extracting the organic layer. The final concentrations of HZTF or VC in the reaction system are 30.00, 60.00, 90.00, 120.00, and 150.00 *μ*g/ml. The absorbance was measured at 532 nm by the ultraviolet-visible spectrophotometer. The decrease of absorbance indicates the increase of antioxidant activity of the samples. The antioxidant activity value of the samples was expressed as the percentage of lipid peroxidation inhibition rate which was calculated by the following equation:

(2) $$\begin{array}{c} \text{inhibition effect }\left(\%\right)=\left(1-\frac{A_{\text{S}}}{A_{\text{C}}}\right)\times 100, \end{array}$$

where *A*_S_ is the absorbance of test sample and *A*_C_ is the absorbance of blank control.

#### 2.7.2. DPPH^·^ Radical-Scavenging Ability

DPPH^·^ radical-scavenging capacity was determined according to the modified method by Luo et al. [26]. HZTF and VC samples were prepared in 70% ethanol at the difference concentrations, then 1 ml of each sample mixed with 1 ml DPPH solution (0.1 M in 50% ethanol) and 3 ml distilled water. The final concentrations of HZTF are 4.00, 12.00, 20.00, 28.00 and 36.00 *μ*g/ml, and VC are 2.00, 4.00, 6.00, 8.00 and 10.00 *μ*g/ml. The mixtures were vortexed and allowed to equilibrate for 20 min at room temperature in the dark, and then measured the absorbance at 517 nm. [27, 28]. Scavenging activity was calculated using the formula:

(3) $$\begin{array}{c} \text{scavenging effect }\left(\%\right)=\left(1-\frac{A_{\text{S}}}{A_{\text{C}}}\right)\times 100, \end{array}$$

where *A*_S_ is the absorbance of the samples at different concentration and *A*_C_ is the absorbance of control.

#### 2.7.3. ABTS^+^ Radical-Scavenging Capacity

ABTS radical cation (ABTS^+^ radical)-scavenging capacity was carried out according to the method of Re et al. with modification according to the situation in our laboratory [29–31]. ABTS^+^ stock solution (3.0 mg/ml) was prepared by dissolving ABTS in 2.5 mM potassium persulfate solution and incubated the mixture in dark overnight at room temperature. The ABTS^+^ solution was diluted with sodium phosphate buffer (pH 7.4) to obtain absorbance of 0.7 ± 0.1 at 734 nm. 4 ml of HZTF or VC in different concentrations were mixed with 4 ml of ABTS^+^ radical working solution and reacted for 300 s. Then, the absorbance was measured at 734 nm. The final concentrations of HZTF were 5.00, 7.50, 10.00, 12.50, and 15.00 *μ*g/ml, and VC were 1.00, 2.00, 3.00, 4.00, and 5.00 *μ*g/ml.

(4) $$\begin{array}{c} {\text{ABTS}}^{+}\text{ radical scavenging }\left(\%\right)=\left(1-\frac{A_{\text{S}}}{A_{\text{C}}}\right)\times 100, \end{array}$$

where *A*_S_ is the absorbance of sample and *A*_C_ is the absorbance of the black control.

### 2.8. Cell-Base *In Vitro* Studies

#### 2.8.1. Cell Culture

RAW murine macrophage 264.7 (RAW264.7) and THP-1 cell lines were purchased from the Institute of Biochemistry and Cell Biology, CAS, Shanghai, China. The RAW264.7 were cultured in Dulbecco's modified eagle's medium (DMEM) with 10% fetal bovine serum (FBS), 1x antibiotic solution (streptomycin (100 U/ml) and penicillin (100 U/ml)) in a humidified atmosphere supplied with 5% CO_2_ and maintained at a temperature of 37°C. Cells were allowed to grow till they reached a confluency of 80–90% and washed with phosphate buffered saline (PBS) with regular replacement of culture medium.

THP-1 cells were maintained in 1640 medium supplemented with 10% FBS in a humidified atmosphere supplied with 5% CO_2_ and maintained at temperature of 37°C. Cells were allowed to grow till they reached a density of 2 × 10^6^ and then added the same volume of culture medium.

#### 2.8.2. Cell Viability

Cell viability was performed by MTT assay. RAW264.7 macrophage cells were seeded in a 96-well culture plate. THP-1 cells were seeded in another 96-well culture plate for 3 hours with 30.82 *μ*g/ml PMA, the day before drug treatment. After 24 h incubation, the cells were stimulated with varying concentrations of HZTF (3.12, 6.25, 12.50, 25.00, 50.00, 100.00, and 200.00 *μ*g/ml) in FBS-free medium for further 24 h. After incubation, 10.0 *μ*L MTT DMSO solutions (5 mg/ml) were added to each well. Then, the plates were incubated for 4 h at 37°C in dark. After incubation, 90 *μ*L medium was removed from each well, 100 *μ*L of DMSO was added to the wells, and then the plates were incubated for 15 min at 37°C under gentle shaking to dissolve the tetrazolium dye. The cell viability was calculated according to the results of absorbance at 570 nm and expressed as relative percentage viability. The relative cell percentage viability was calculated by the following equation:

(5) $$\begin{array}{c} \text{cell viability }\left(\%\right)=\left[\frac{A_{\text{S}}-A_{\text{b}}}{A_{\text{C}}-A_{\text{b}}}\right]\times 100, \end{array}$$

where *A*_S_ is the absorbance of HZTF, *A*_C_ is the absorbance of the control, and *A*_b_ is the absorbance of cell blank.

#### 2.8.3. Cell Treatment

Cells reaching a concentration of 5 × 10^6^ cells/well in 12-well culture plate were considered for drug treatment. RAW264.7 macrophages were treated with varying concentrations of HZTF (100.00 and 200.00 *μ*g/ml) and H_2_O_2_ (882.09 nM) for 24 h and prepared HZTF DMSO working solution in different concentrations. THP-1 cells were seeded with 30.82 *μ*g/ml PMA in a 24-well culture plate for 3 hours the day before drug treatment. After 24 h of incubation, the cells were stimulated with varying concentrations of HZTF (6.25, 12.50, 25.00 and 50.00 *μ*g/ml) and MSU in FBS-free medium for further 8 h, and then the cell culture medium of each well was collected for Elisa analysis.

#### 2.8.4. Quantification of mRNA Levels by Quantitative Real-Time PCR

Total RNA was isolated from RAW264.7 macrophages treated with various concentrations of DEX (25 *μ*g/ml) and HZTF (100.00 and 200.00 *μ*g/ml) with H_2_O_2_ (0.15%) stimulation for 24 h and THP-1 cells pretreated with HZTF (25.00 and 50.00 *μ*g/ml) and stimulated by MSU (1 mg/ml) for 8 h using the TRIzol reagent according to manufacturer's instructions. The total RNA was reverse transcribed by using cDNA reverse transcriptase kit, and then mRNA expression was amplified by PCR mastermix. Gene specific primers were designed manually using online NCBI primer-BLAST tool. Primer sequences of forward and reverse are shown in Table 1. Quantitative real-time PCR (qRT-PCR) was used to analyse the IL-1*β*, caspase-1, NLRP3, NQO1, and GAPDH gene expression of RAW 264.7 and IL-1*β* of THP-1, respectively. Transcription levels were assessed to utilize the step on real-time thermal cycler with PCR mastermix. Thermal cycling conditions were as follows: denaturation at 95°C for 5 s, annealing at 60°C for 30 s, and extension at 65°C for 5 s. The levels of GAPDH gene expression served as an internal control.

#### 2.8.5. ELISA Analysis

THP-1 cells were pretreated with MCC950 (4.26 *μ*g/ml) or HZTF (6.25, 12.50, 25.00 and 50.00 *μ*g/ml), stimulated by MSU (1 mg/ml) for 8 h, and collected the supernatant. Secretion of inflammatory cytokine (IL-1*β*, IL-6 and TNF-*α*) was measured by ELISA kits according to the assay procedure.

#### 2.8.6. Western Blotting Analysis

Protein samples were obtained from the culture medium as same as in the ELISA assay and concentrated by the ultrafiltration device. Protein sample was denatured by mixing with 1/4 volume of 5x loading buffer, and metal bath at 100°C for 10 min. Samples were resolved by electrophoresis with 10% SDS-PAGE and transferred onto a PVDF membrane (Merk, Germany). After blocking nonspecific binding sites for 2 h with 5% dried skim milk dissolved in TBST, the membranes were individually incubated for overnight with anti-IL-1*β*, anti-IL-6, anti-caspase-1, anti- PGE2, and ASC. Develop the color of the blot rocking in 3, 3′-diaminobenzidine (DAB) substrate solution. Stop the reaction by pouring out the substrate after the expected band appears and then well rinsed with distilled water repeatedly. Dry the membrane, and store it in the dark place.

#### 2.8.7. Confocal Microscopic Analysis

THP-1 cells were pretreated with MCC950 (4.26 *μ*g/ml) or HZTF (50.00 *μ*g/ml) and stimulated by MSU (1 mg/ml) for 8 h. Briefly, THP-1 cells were plated on coverslips and incubated with an anti-ASC antibody and then incubated with an anti-rabbit IgG-FITC antibody. Recording with a laser scanning confocal microscope (LSM710, Carl Zeiss, Oberkochen, Germany), the ASC speck formation was analyzed using Zen2010 software.

### 2.9. Statistical Analysis

All results were expressed as mean ± standard deviation (*n* = 3). Statistical analysis was carried out by SPSS 21 statistical software (IBM Inc., USA). A statistical difference was considered significant when *P*-values are less than 0.05.

## 3. Results

### 3.1. Quality Standard of HZTF by HPLC

**Figure 1:**
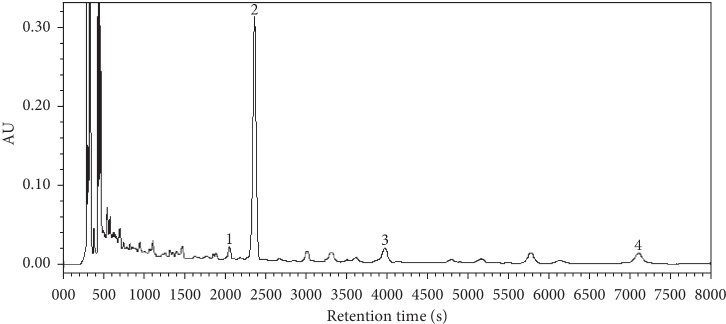
The quality standard of HZTF was determined using an HPLC system, plantamajoside (1), polygonum cuspidin (2), specnuezhenide (3) and resveratrol glucoside (4).

As shown in Figure 1, four substances in HZTF were selected as content indicators: (1) plantamajoside, (2) polygonum cuspidin, (3) specnuezhenide, and (4) resveratrol glucoside, and all of them had distinct peaks under 280 nm ultraviolet absorption.

### 3.2. Effect of HZTF and Tongfengshu in *In Vivo* Studies

To determine the degree of edema, the swelling value of the right posterior foot ankle joints of rats were measured (Table 2). The foot-swelling values of MSU crystal-induced rats were significantly higher than that of the control rats at 1 h to 7 h (*P* < 0.05). Paw edema decreased significantly in MSU crystal-induced rats treated with HZTF (0.50 and 1.00 g/kg body weight) at 1 h to 7 h and Tongfengshu (1.50 g/kg body weight) at 1 h to 7 h (*P* < 0.05).

**Figure 2:**
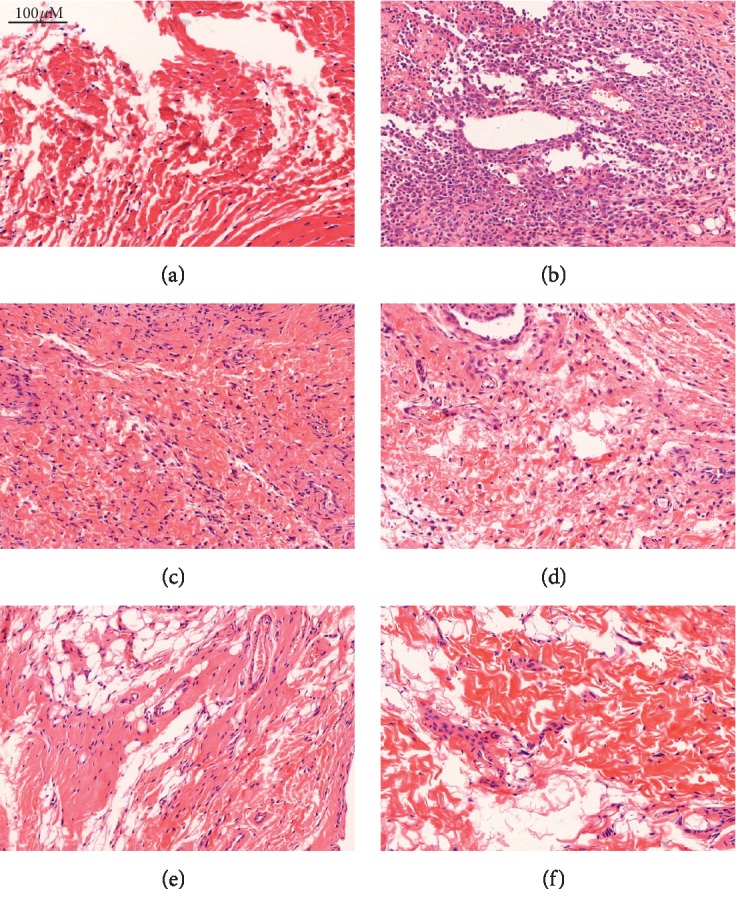
Histological examination of rat joints injected with MSU crystals (magnification, ×200). In the control group, synovial membrane was normal and no inflammatory cell infiltration was found. (a) Joints injected with MSU crystals demonstrated leukocytes infiltrations and edema. Synovial tissue proliferation of nodularity was observed in the synovial lining of the joint, as well as synovial membrane cells with some zones of degeneration and necrosis (MSU alone). (b) Acute inflammation with polymorphonuclear infiltration in the synovial lining of MSU crystal-injected rats was attenuated by Tongfengshu and HZTF. (c) The Tongfengshu (1.50 g/kg) group has no obvious edema in loose connective tissue, but there were still demonstrated inflammatory cells infiltration. (d–f) HZTF (0.25, 0.5 and 1.00 g/kg) groups demonstrated only mild infiltration of scattered leukocytes. The synovial of HZTF group (MSU and HZTF 1.00 g/kg) appeared normal.

To compare the histological changes in the joints between different groups, joint tissues were sectioned and examined via H&E staining. The coverage of synovial cells on synovial cavity of the joints was fragmentary, and there was edema in loose connective tissue, as well as local infiltration of leukocytes (Figure 2). However, rats treated with HZTF (0.50 and 1.00 g/kg body weight) and Tongfengshu (1.50 g/kg body weight) decreased edema and leukocyte infiltration after 7 h. This change correlated well with the decrease in paw swelling induced by HZTF and Tongfengshu treatment.

### 3.3. *In Vitro* Cell-Free Antioxidation Experiment of HZTF

**Figure 3:**
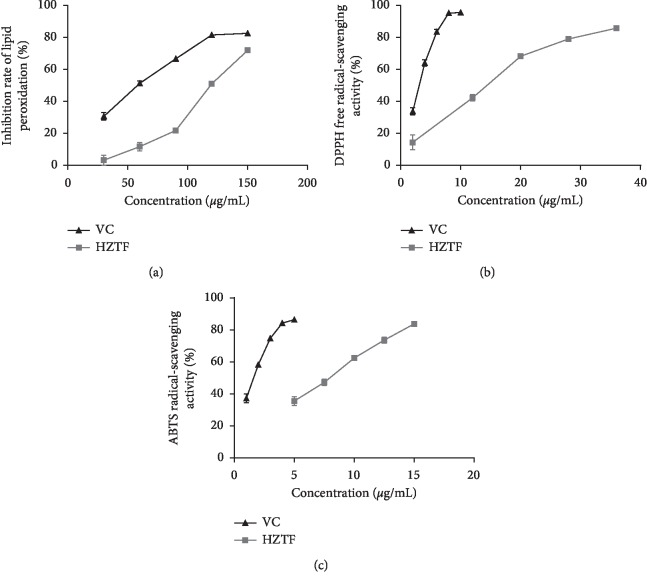
Lipid peroxidation inhibition ability. (a) DPPH^·^ free radical-scavenging activity and (b) ABTS^+^ radical-scavenging capacity (c) of HZTF at different concentrations.

To investigate whether the antigout effect of HZTF is due to its antioxidant activity, we measured the antioxidant activity of HZTF using multiple methods. As shown in Figure 3(a), we can know that HZTF can significantly inhibit lipid peroxidation when the concentration was higher than 60.00 *μ*g/ml, and the IC_50_ values of VC and HZTF are 49.94 ± 3.67 and 114.62 ± 3.43 *μ*g/ml, respectively, which mean the lipid peroxidation inhibition ability of HZTF is significantly weaker than VC (*P* < 0.05). It is observed that the DPPH^·^ radical-scavenging activity of HZTF is shown in Figure 3(b). The IC_50_ values of VC and HZTF are 2.81 ± 0.62 and 13.04 ± 2.80 *μ*g/ml, respectively. Though it can inhibit the DPPH^·^ radical, the IC_50_ value of HZTF is significantly higher than VC (*P* < 0.05). As shown in Figure 3(c), the ABTS^+^ radical-scavenging activity of both VC and HZTF are in dose-effect relationships. HZTF can scavenge the ABTS^+^ radical significantly when the concentration was higher than 5.00 *μ*g/ml and the IC_50_ values of VC and HZTF are 1.48 ± 0.70 and 7.52 ± 0.50 *μ*g/ml, respectively, which means the ABTS^+^ radical-scavenging activity of HZTF is significantly weaker than VC (*P* < 0.05).

### 3.4. Cell Viability

**Figure 4:**
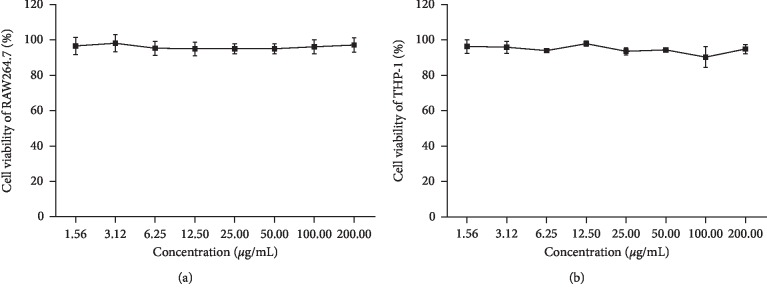
Cell viability of RAW264.7 (a) and THP-1 (b) cells treated with HZTF at different concentrations.

MTT assay was utilized to measure the cytotoxicity of HZTF on RAW264.7 macrophages and THP-1 cells.  Figure 4 shows that the cell viability was not significantly altered after treating with HZTF for 24 h up to 200.00 *μ*g/ml concentration, suggesting no cytotoxicity of HZTF up to 200.00 *μ*g/ml.

### 3.5. Experiment with RAW264.7 Macrophages

#### 3.5.1. RT-qPCR Analysis

**Figure 5:**
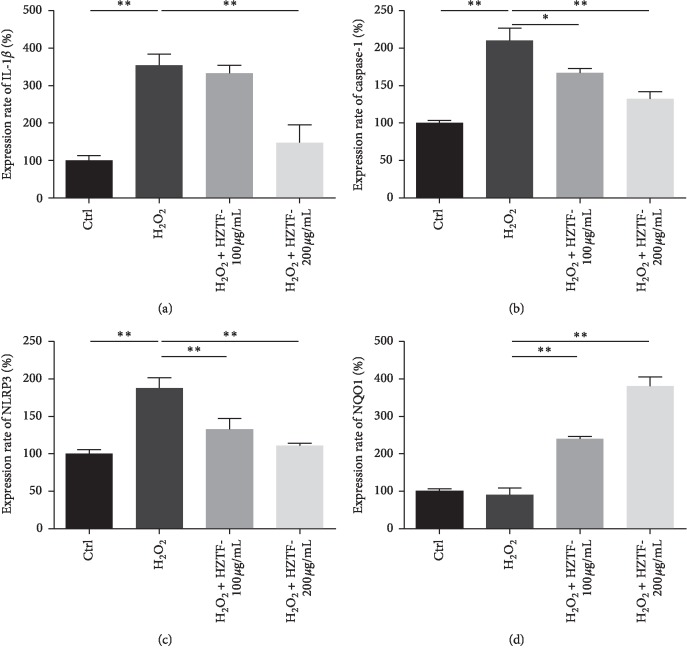
Effect of HZTF on IL-1*β* (a), caspase-1 (b), NLRP3 (c), and NQO1 (d) expression in H_2_O_2-_stimulated RAW264.7 macrophages. The values are expressed as mean ± SD. Comparisons are made with: control or HZTF (100, 200 *μ*g/ml) treated versus H2O2-stimulated RAW264.7 macrophages. ^*∗*^means significantly different (*P* < 0.05) and ^*∗∗*^means very significantly different (*P* < 0.01).

As shown in Figure 5, changes of IL-1*β*, caspase-1, NLRP3, and NQO1 expression in H_2_O_2_-induced RAW264.7 macrophages were compared with the control group without H_2_O_2_, and the expression of IL-1*β*, caspase-1, and NLRP3 in RAW264.7 macrophages was increased by treatment with 0.15% H_2_O_2_ for 24 h. Interestingly, the expression of IL-1*β*, caspase-1, and NLRP3 in RAW264.7 macrophages treated with HZTF at 100.00 and 200.00 *μ*g/ml were significantly reduced (*P* < 0.05). Moreover, an obvious difference (*P* < 0.05) was found between the mRNA expression of NQO1 with or without HZTF (100.00 and 200.00 *μ*g/ml), and the expression of NQO1 was increased with the increase of HZTF concentrations, which may indicate a dose-dependent relationship between NQO1 expression and HZTF concentrations. In conclusion, the results showed that H_2_O_2_ upregulated the expression of IL-1*β*, caspase-1, and NLRP3 and downregulated the expression of NQO1. Treatment with HZTF inhibits the expression of IL-1*β*, caspase-1, and NLRP3 and enhanced the expression of NQO1 in H_2_O_2_-induced RAW264.7 macrophages at the same time.

### 3.6. Experiment with THP-1 Cells

#### 3.6.1. ELISA Analysis

**Figure 6:**
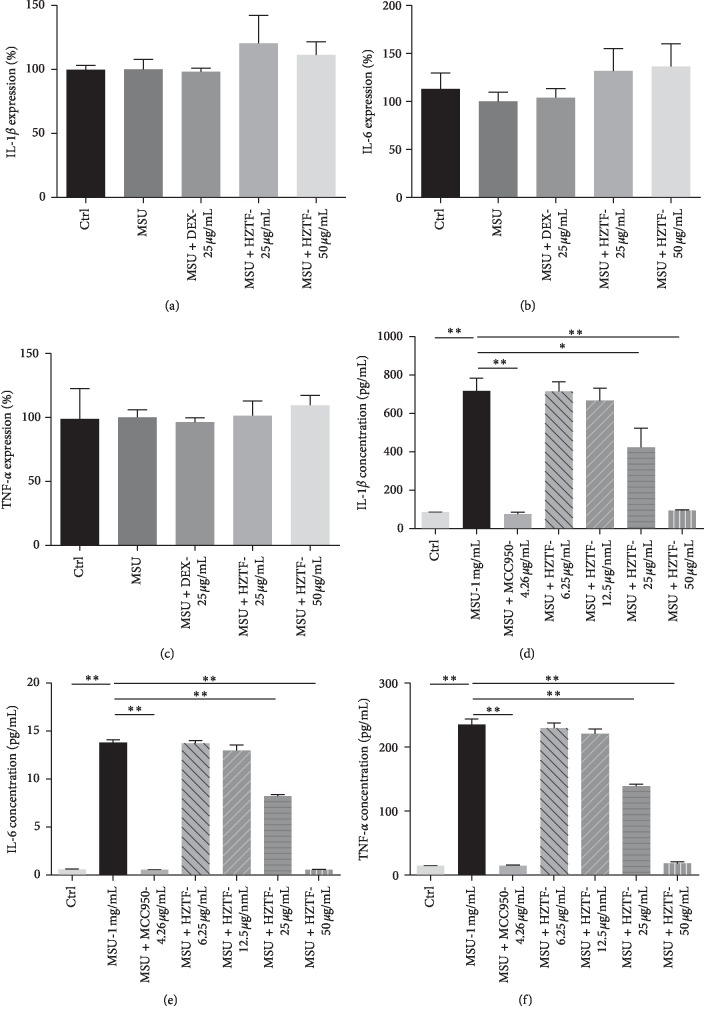
Effect of HZTF on IL-1*β* (a), IL-6 (b), and TNF-*α* (c) expression and IL-1*β* (d), IL-6 (e), and TNF-*α* (f) secretion in THP-1 stimulated by MSU. ^*∗*^means significant different (*P* < 0.05) and ^*∗∗*^means very significant different (*P* < 0.01).

To investigate the mechanism of HZTF against gout, qPCR was used to measure the effect of HZTF on IL-1*β*, IL-6, and TNF-*α* expression and ELISA was used to measure the effect of HZTF on IL-1*β*, IL-6, and TNF-*α* secretion. As shown in Figures 6(a)–6(c) MSU or HZTF does not significantly affect the expression of IL-1*β*, IL-6, and TNF-*α*. As shown in Figures 6(d)–6(f), HZTF significantly reduced the secretion of IL-1*β*, IL-6, and TNF-*α* in THP-1 cells stimulated by MSU (*P* < 0.05). The IC_50_ of IL-1*β*, IL-6, and TNF-*α* secretion in THP-1 cells stimulated by MSU were 20.94 ± 2.13, 26.04 ± 1.28, and 24.87 ± 0.68 *μ*g/ml, respectively.

### 3.7. Western Blotting Analysis

**Figure 7:**
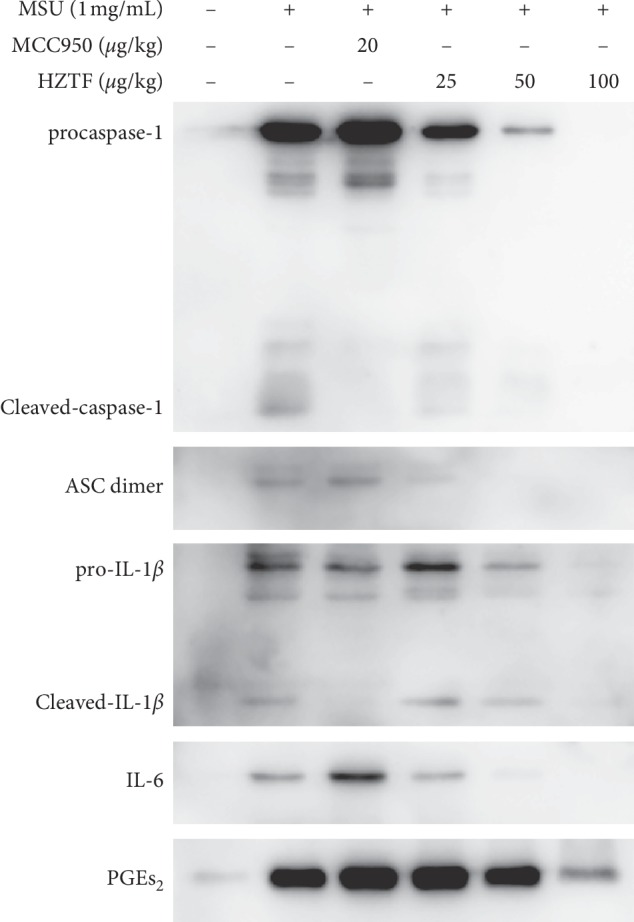
Effect of HZTF on procaspase-1, cleaved-caspase-1, pro-IL-1*β*, cleaved-IL-1*β*, ASC dimer, IL-6, and PGE_2_ protein expression secreted into culture medium.

As shown in Figure 7 HZTF inhibited the procaspase-1, cleaved-caspase-1, pro-IL-1*β*, cleaved-IL-1*β*, ASC dimer, IL-6, and PGEs_2_ in the culture medium which were secreted by THP-1 cells in a dose-dependent manner.

### 3.8. Confocal Microscopic Analysis

**Figure 8:**
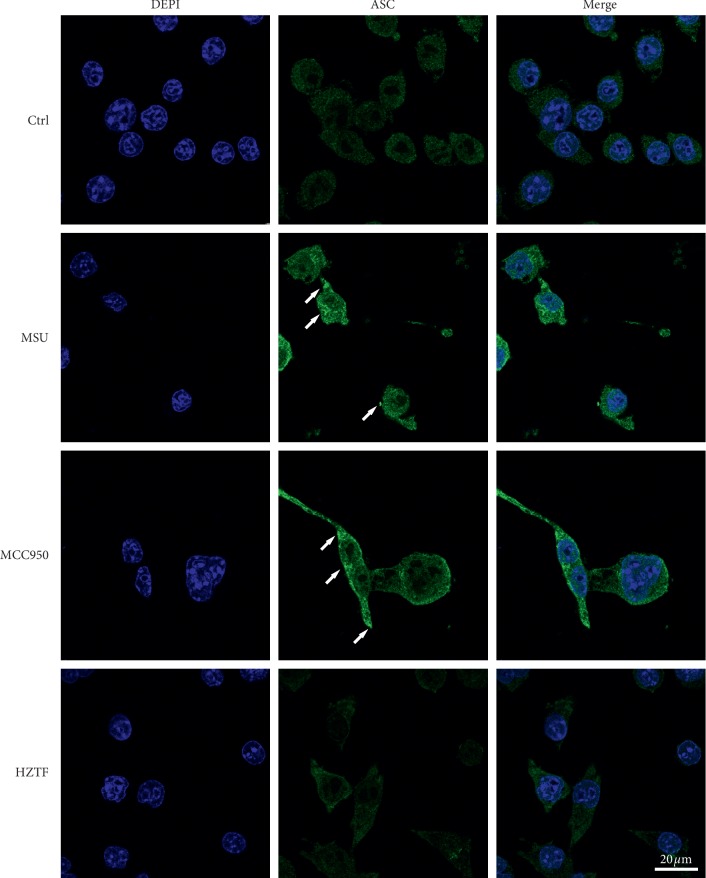
THP-1 cells were fixed, permeabilized, and stained for ASC (green), and the nuclei were stained with 4′,6-diamidino-2-phenylindole (DAPI; blue). The arrows indicate ASC speckles (Magnification, ×600).

As a symbol of inflammasome activation, ASC forms oligomers in response to NLRP3 activators. Confocal microscopy analysis intuitively showed that HZTF suppressed MSU crystal-induced formation of ASC speckles in THP-1 cells (Figure 8).

## 4. Discussion

Inflammatory response to MSU crystals triggered the acute symptoms of gout and mainly mediated by macrophages and neutrophils. Intra-articular injection of MSU crystal can simulate gouty arthritis in humans and produces a painful response which is similar to the acute gouty attacks occurrence spontaneously [32]. Significant swelling accompanied by an extensive inflammatory response after MSU injection in the ankle joint is an important symbol in determining the degree of inflammation and therapeutic efficacy. The primary pathological symbol of gout is the neutrophils accumulated and infiltrated in the joint tissues, which actively phagocytosed MSU crystals. Then, the membrane lysed and released inflammatory cytokines and free radicals amplified the inflammatory response [33, 34]. We observed similar changes in the joint MSU injection rat model, so this model is suitable for studying the pathological mechanism of gout arthritis.

Plants which give antioxidant activities should be highly potent in the management of gout because they often share xanthine oxidase (XO) inhibitory effects [35, 36]. We measured the antioxidant activities to study whether the antigout effect of HZTF is related to its antioxidant capacity. Lipid peroxidation occurs in the process of reactive oxygen species (ROS) oxidizing the biological membranes, which ROS reacts with membrane phospholipids, enzyme and membrane receptor-associated polyunsaturated side chain, and nucleic acid fatty acid and produces lipid peroxidation products such as malondialdehyde and 4-hydroxynonenal, and then the fluidity and permeability of cell membrane changed, resulting in the change of structure and function of cells [37, 38]. The free radical-scavenging capacity of HZTF was measured using commercially available stable free radical DPPH^·^. HZTF presented DPPH^·^ radical-scavenging capacity to some extent. It appears that antioxidant activity and reduction of DPPH^·^ both require the presence of a free OH group on the ellipticine ring [39], and if HZTF can reduce DPPH^·^, it may suggest that HZTF contains compounds of similar structure. Based on the special chemical properties of formed free radicals, ABTS assay is frequently used to measure the radical-scavenging capacity of compounds [40]. The ABTS^+^ radical elimination is a common antioxidant method as well as the DPPH^·^ method to evaluate the antioxidant capacity of the chemical compound. Based on previous descriptions, *Polygonum cuspidatum*, *Plantain*, and *Ligustrum*, which were used to produce HZTF, can extract compounds with good antioxidant activity, and similar to HZTF, these compounds were also extracted from ethanol, which suggest that the main antioxidant compounds containing HZTF may be phenolic compounds too [41–43]. According to the results, the lipid peroxidation inhibition ability, DPPH^·^ radical-scavenging ability and ABTS^+^ radical-scavenging capacity of HZTF were significantly weaker than VC (*P* < 0.05). VC is a strong antioxidant [44], but seldom used in acute gout treatment. HZTF has weaker antioxidant capacity than vitamin C, but has a strong antigout capacity, so we believed that the antioxidant capacity of HZTF may not be the main mechanism of its antigout effect.

IL-1*β* is a proinflammatory cytokine that induces local and systemic inflammation aimed to eliminate foreign matters and microorganisms. However, exorbitant levels of inappropriate IL-1*β* production have been shown to be a key process in the etiology of the disease. In these conditions, blocking IL-1*β* has proven very effective in clinical studies [45]. Caspase-1 and NLRP3 are closely linked to the pathogenesis of various metabolic diseases including gouty arthritis [22, 46]. At the same time, the expression increase of IL-1*β*, caspase-1, and NLRP3 is closely related to oxidative stress, and if we reduce the level of oxidative stress, we may suppress the expression of IL-1*β*, caspase-1, and NLRP3 [47]. NQO1 is an important cellular antioxidant enzyme, and NQO1 is an Nrf2-regulated downstream enzyme, and the mRNA expression of NQO1 was observed after treatment. In the present study, we investigated whether HZTF has any regulatory effect on the gene expression of IL-1*β*, caspase-1, NLRP3, and NQO1 in H_2_O_2_ stimulated RAW264.7 macrophages. Changes of IL-1*β*, caspase-1, and NLRP3 expression in H_2_O_2_-induced RAW264.7 macrophages were compared with the control group without H_2_O_2_; the expression of IL-1*β*, caspase-1, and NLRP3 in RAW264.7 macrophages was increased by treatment with 0.15% H_2_O_2_ for 24 h. Interestingly, the expression of IL-1*β*, caspase-1, and NLRP3 in RAW264.7 macrophages treated with HZTF at 100.00 and 200.00 *μ*g/ml were significantly reduced (*P* < 0.05). Furthermore, an obvious difference (*P* < 0.05) was found between the mRNA expression of NQO1 with or without HZTF (100.00 and 200.00 *μ*g/ml), the expression of NQO1 was increased with the increase of HZTF concentrations, which may indicate a dose-dependent relationship between NQO1 expression and HZTF concentrations. In conclusion, the results showed that H_2_O_2_ upregulated the expression of IL-1*β*, caspase-1, and NLRP3, and downregulated the expression of NQO1. Treatments with HZTF inhibit the expression of IL-1*β*, caspase-1, and NLRP3, and enhanced the expression of NQO1 in H_2_O_2_-induced RAW264.7 macrophages at the same time.

Development of the acute and chronic inflammatory responses known as gout and pseudogout are associated with the deposition of MSU crystals, in joints and periarticular tissues. MSU engage the caspase-1-activating NALP3 (also called cryopyrin) inflammasome, resulting in the production of active IL-1*β* [22]. Macrophages from mice deficient in components of the inflammasome such as ASC (RAW264.7 macrophages) are defective in crystal-induced IL-1*β* activation [48]. THP-1 cells were chosen for this experiment. According to the result, MSU crystals did not increase IL-1*β* mRNA expression, but increased IL-1*β* secretion, and HZTF also did not affect the mRNA expression of IL-1*β*, IL-6, and TNF-*α*, but significantly reduced the secretion of IL-1*β*, IL-6, and TNF-*α* in THP-1 cells stimulated by MSU. In the western blotting test, we can know that HZTF inhibited procaspase-1, cleaved-caspase-1, pro-IL-1, cleaved-IL-1*β*, and IL-6 at the same time, suggesting that HZTF may inhibit not only the inflammasomes but also the NF-*κ*b signaling. Also, in the western blotting result, HZTF showed inhibition effect in PGE_2_ secretion, which suggests that the mechanism of antigout effect of HZTF may be varied. At the same time, confocal microscopy analysis intuitively demonstrated the inhibitory effect of HZTF on ASC dimerization. These results suggested that HZTF may prevent gout by inhibiting NALP3 inflammasome as well as blocking the NF-*κ*b signaling pathway, which will increase the expression of IL-1*β*, IL-6, and TNF-*α* [49].

Earlier reports indicated that four flavours of traditional Chinese medicinal materials which were used to produce HZTF contained several components, of which plantamajoside, *Polygonum cuspidatum*, specnuezhenide and resveratrol glucoside were found to be the main active constituent [2–11]. In present study, HPLC fingerprint analysis of HZTF showed distinct peaks of all four active constituents under 280 nm ultraviolet absorption.

## 5. Conclusion

The present study confirmed the antigout effects of HZTF in *in vivo* experiments and showed its reduction effects on the secretion of proinflammatory cytokines IL-1*β*, IL-6, and TNF-*α*. At the same time, whether its antigout effect is related to its antioxidant capacity needs further study. This study provides a new promising therapeutic TCM for gout, and the precise mechanism of action is worth further to be investigated.

## Figures and Tables

**Table 1:** Sequences of PCR primers used for qRT-PCR.

Species	Gene	Primer	Sequence
*Mice*	IL-1*β*	Forward primer	ACCTGGGCTGTCCTGATGAGAG
		Reverse primer	TGTTGATGTGCTGCTGCGAGAT
	Caspase-1	Forward primer	GGGACCCTCAAGTTTTGCC
		Reverse primer	CAACTTGAGCTCCAACCCTC
	NLRP3	Forward primer	TCTGTGTGGACCTAAGCCCC
		Reverse primer	GGGATACAGCCTTTCTCGGG
	NQO1	Forward primer	GAGAGGATGGGAGGTACTC
		Reverse primer	AATATCTGGGCTCAGGCGTC
	GAPDH	Forward primer	GTCATTGAGAGCAATGCCAG
		Reverse primer	GTGTTCCTACCCCCAATGTG

*Human*	IL-1*β*	Forward primer	TGATGGCTTATTACAGTGGCAATG
		Reverse primer	GTAGTGGTGGTCGGAGATTCG
	IL-6	Forward primer	CACTGGTCTTTTGGAGTTTGAG
		Reverse primer	GGACTTTTGTACTCATCTGCAC
	TNF-*α*	Forward primer	CAGAGGGAAGAGTTCCCCAG
		Reverse primer	CCTTGGTCTGGTAGGAGACG
	GAPDH	Forward primer	AGCCTCAAGATCAGCAATG
		Reverse primer	CACGATACCAAAGTTGTCATGGAT

**Table 2:** Effect of HZTF and Tongfengshu on the ankle joint edema of MSU crystal-induced rats.

Treatment	Dose (g/kg)	*n*	Foot-swelling values (cm, mean ± SD)						
			1 h	2 h	3 h	4 h	5 h	6 h	7 h
Ctrl	—	10	0.009 ± 0.002^*∗∗*^	0.010 ± 0.004^*∗∗*^	0.008 ± 0.003^*∗∗*^	0.0010 ± 0.003^*∗∗*^	0.004 ± 0.002^*∗∗*^	0.005 ± 0.001^*∗∗*^	0.003 ± 0.000^*∗∗*^
MSU alone	—	10	0.169 ± 0.055	0.185 ± 0.056	0.208 ± 0.065	0.254 ± 0.059	0.289 ± 0.058	0.323 ± 0.051	0.304 ± 0.061
Tongfengshu	1.50	10	0.107 ± 0.023^*∗*^	0.142 ± 0.046^*∗*^	0.161 ± 0.042^*∗*^	0.193 ± 0.049^*∗*^	0.202 ± 0.048^*∗*^	0.253 ± 0.053^*∗*^	0.239 ± 0.055^*∗*^
HZTF	0.25	10	0.139 ± 0.035	0.178 ± 0.050	0.197 ± 0.051	0.206 ± 0.056^*∗*^	0.226 ± 0.055^*∗*^	0.257 ± 0.028	0.247 ± 0.065^*∗*^
HZTF	0.50	10	0.123 ± 0.042^*∗*^	0.150 ± 0.056^*∗*^	0.168 ± 0.056^*∗*^	0.204 ± 0.060^*∗*^	0.218 ± 0.041^*∗*^	0.245 ± 0.025^*∗*^	0.237 ± 0.062^*∗*^
HZTF	1.00	10	0.121 ± 0.048^*∗*^	0.135 ± 0.044^*∗*^	0.150 ± 0.042^*∗*^	0.179 ± 0.037^*∗*^	0.197 ± 0.038^*∗∗*^	0.217 ± 0.034^*∗∗*^	0.189 ± 0.050^*∗∗*^

^*∗*^ *P* < 0.05 when compared with MSU groups, ^*∗∗*^*P* < 0.01 when compared with MSU groups.

## Data Availability

The data used to support the findings of this study are available from the corresponding author upon request.

## Abbreviations

TCM: Traditional chinese medicine
MSU: Monosodium urate
HZTF: Huzhentongfeng
VC: Ascorbic acid
DEX: Dexamethasone
DMEM: Dulbecco's modified eagle's medium
FBS: Fetal bovine serum
PBS: Phosphate-buffered saline
MTT: Methylthiazolyldiphenyl-tetrazolium bromide
PMA: Phorbol-12-myristate-13-acetat
DPPH: 1,1-Diphenyl-2-picryl-hydrazyl
ABTS: 3-Ethylbenzthiazoline-6-sulfonic acid
AAPH: 2,2′-Azobis (2-methylpropionamidine) dihydrochloride
Caspase-1: Cysteine-aspartic proteases-1
NLRP3: NACHT, LRR, and PYD domains-containing protein 3
ASC: Apoptosis-associated speck-like protein containing
Caspase-1: Cysteinyl aspartate specific proteinase-1
IL-1*β*: Interleukin-1*β*
IL-6: Interleukin-6
TNF-*α*: Tumor necrosis factor-*α*
PGE_2_: Prostaglandin E2
NF-*κ*B: Nuclear factor-*κ*b
NQO1: NADPH dehydrogenase, quinone-1
Nrf2: Nuclear factor (erythroid-derived 2)-like 2
HPLC: High-performance liquid chromatography
Elisa: Enzyme-linked immunosorbent assay
IC_50_: Half maximal inhibitory concentration
H&E: Hematoxylin and eosin
SD: Standard deviation
ANOVA: One-way analysis of variance
LPS: Lipopolysaccharide
PCR: Polymerase chain reaction
RT-qPCR: Real-time quantitative polymerase chain reaction.
